# Impact assessment of malaria vector control using routine surveillance data in Zambia: implications for monitoring and evaluation

**DOI:** 10.1186/1475-2875-11-437

**Published:** 2012-12-29

**Authors:** Emmanuel Chanda, Michael Coleman, Immo Kleinschmidt, Janet Hemingway, Busiku Hamainza, Freddie Masaninga, Pascalina Chanda-Kapata, Kumar S Baboo, David N Dürrheim, Marlize Coleman

**Affiliations:** 1 Ministry of Health, National Malaria Control Centre, P.O. Box 32509, Lusaka, Zambia; 2 Liverpool School of Tropical Medicine, Pembroke Place, Liverpool, L3 5QA, UK; 3 MRC Tropical Epidemiology Group, London School of Hygiene and Tropical Medicine, Keppel St, London, WC1E 7HT, UK; 4 World Health Organization, WHO Country Office, Lusaka, Zambia; 5 University of Zambia, School of Medicine, P.O. Box 50110, Lusaka, Zambia; 6 Ministry of Heath, Headquarters, Ndeke House, P.O. Box 30205, Lusaka, Zambia; 7 Health Protection - Hunter Medical Research Institute, New South Wales, Australia

## Abstract

**Background:**

Malaria vector control using long-lasting insecticidal nets (LLINs) and indoor residual spraying (IRS), with pyrethroids and DDT, to reduce malaria transmission has been expansively implemented in Zambia. The impact of these interventions on malaria morbidity and mortality has not previously been formally assessed at the population level in Zambia.

**Methods:**

The impact of IRS (15 urban districts) and LLINs (15 rural districts) implementation on severe malaria cases, deaths and case fatality rates in children below the age of five years were compared. Zambian national Health Management Information System data from 2007 to 2008 were retrospectively analysed to assess the epidemiological impact of the two interventions using odds ratios to compare the pre-scaling up year 2007 with the scaling-up year 2008.

**Results:**

Overall there were marked reductions in morbidity and mortality, with cases, deaths and case fatality rates (CFR) of severe malaria decreasing by 31%, 63% and 62%, respectively between 2007 and 2008. In urban districts with IRS introduction there was a significant reduction in mortality (Odds Ratio [OR] = 0.37, 95% CI = 0.31-0.43, *P* = 0.015), while the reduction in mortality in rural districts with LLINs implementation was not significant (OR = 0.83, 95% CI = 0.67-1.04, *P* = 0.666). A similar pattern was observed for case fatality rates with a significant reduction in urban districts implementing IRS (OR = 0.34, 95% CI = 0.33-0.36, *P* = 0.005), but not in rural districts implementing LLINs (OR = 0.96, 95% CI = 0.91-1.00, *P* = 0.913). No substantial difference was detected in overall reduction of malaria cases between districts implementing IRS and LLINs (*P* = 0.933).

**Conclusion:**

Routine surveillance data proved valuable for determining the temporal effects of malaria control with two strategies, IRS and LLINs on severe malaria disease in different types of Zambian districts. However, this analysis did not take into account the effect of artemisinin-based combination therapy (ACT), which were being scaled up countrywide in both rural and urban districts.

## Background

Malaria remains a major cause of morbidity and mortality in sub-Saharan Africa with at least 75% of deaths in children less than five years of age ascribed to the disease [1]. Most malaria endemic countries are deploying indoor residual spraying (IRS) and/or long-lasting insecticidal nets (LLINs) to combat malaria transmission [2,3]. Measuring the impact of malaria control on reducing disease morbidity and mortality is essential [4] for ensuring the successful implementation of the programme.

Traditionally, the impact of malaria control interventions have been evaluated using repeated population-based surveys to determine parasite prevalence, clinical or laboratory confirmed disease incidence and all-cause mortality [5]. Parasite prevalence in children has generally been the preferred surrogate measure for malaria transmission intensity [6], with routine surveillance data treated with suspicion due to high variability in quality [5].

Recently, efforts have been made to improve routine surveillance data through standardisation of case definitions, collection and collation. Routine health facility data have provided useful insights into the impact of malaria control measures on the incidence of severe malaria [7]. Improved quality surveillance has also proved useful for documenting significant reductions in malaria cases and deaths in all age groups in settings where vector control measures have achieved high community coverage [8-10]. Quality surveillance data have also opened up the opportunity for more thorough geographic mapping of malaria trends to assist in local programme monitoring and resource planning [11].

**Figure 1:**
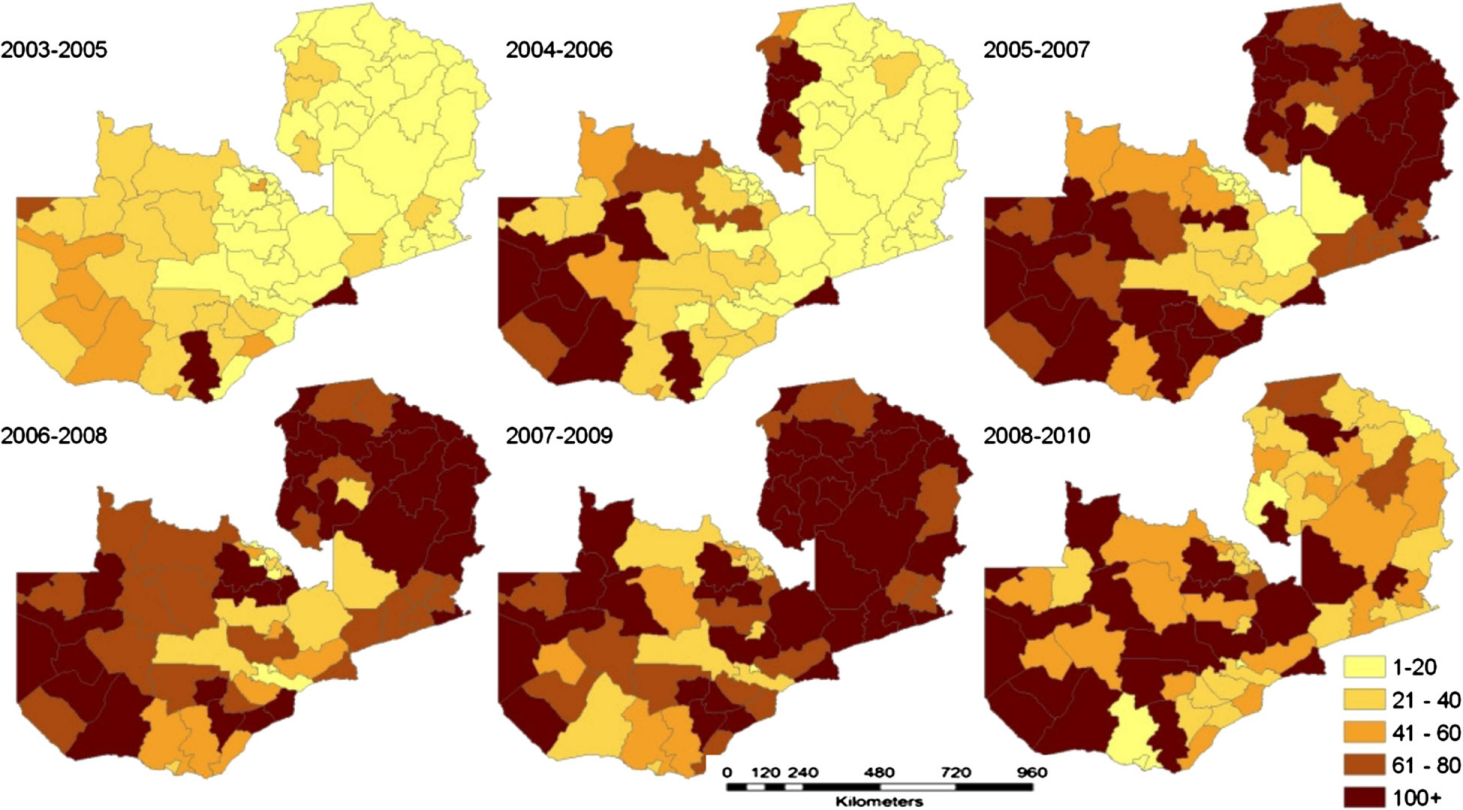
Estimated operational ITN distributions by district in Zambia from 2003–2010, representing percentage of district households receiving 3 ITNs per household (HH) in overlapping 3-year intervals (MoH, 2010).

**Figure 2:**
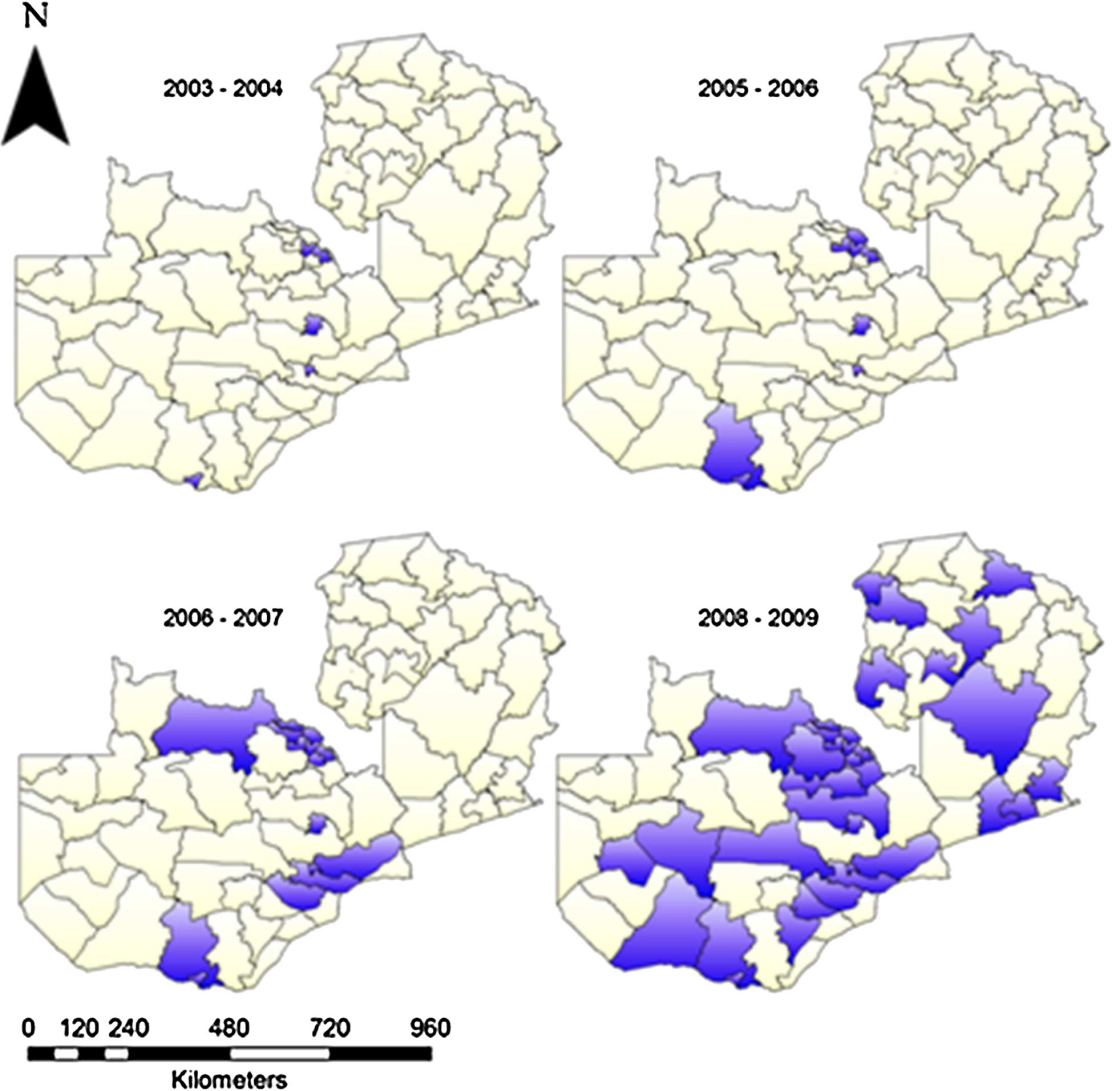
Operational coverage of 36 indoor residual spraying (IRS) districts in Zambia from 2003–2009.

Indoor Residual Spraying (IRS) using pyrethroids and DDT was targeted predominantly at urban and peri-urban areas and LLINs at rural areas. These interventions are being scaled up and monitored by entomological and epidemiological indicators [12,13]. By 2008, 6.1 million LLINs, enough to protect 96% of Zambia’s population, had been distributed country-wide (Figure 1) [14]. Nationally, representative household surveys indicated an increase in household ITN ownership and utilization by children under the age of five years from 43% and 23% in 2006 to 62% and 41% respectively by 2008 (Table 1). Implementation of IRS protected 5.7 million people in 2008 with an average coverage of 90% of over 1.0 million targeted households (Figure 2) [5,14]. The national coverage of both LLINs and IRS has surpassed the international targets of at least 80% of households [15] and it provides a unique opportunity for evaluating the impact of these interventions [16].

**Table 1:** Progress of malaria control in Zambia from 2001 to 2008 (MoH, 2010)

**Indicator**	**DHS 2001/2002**	**MIS 2006**	**DHS 2007**	**MIS 2008**	**χ**^**2 **^*(*2006*–*2008)*	*P (*2006*–*2008)*
Percentage of households with at least one ITN	14	38	53	62	0.0164	0.014
Percentage of households covered with ITN or recent IRS	N/A	43	N.A	66	0.0276	0.032
Percentage of children ages 0–59 months who slept under an ITN the previous night	7	24	29	41	0.0350	0.038
Percentage of children ages 0–59 months with malaria parasitaemia	N/A	22	N/A	10	0.0339	0.035

*Chi-square and P-values, d.f. =1 for 2006 and 2008.

## Methods

Vector control programmes are coordinated and managed by the Zambian Ministry of Health through the National Malaria Control Centre (NMCC). LLINs and IRS are implemented and recorded at district level by the District Health Management Teams (DHMT). Malaria case and hospital malaria specific mortality data from children less than five years of age were obtained from the Zambian national Health Management Information System (HMIS).

### Malaria case and mortality indicator definition

Malaria is diagnosed using either direct microscopy or rapid diagnostic tests (RDTs) in health facilities and generally by the HRP-2 RDT (ICT Malaria Test®, R and R marketing, Cape Town, South Africa) at community level since 2007. The latter is implemented through the Home Management of Malaria (HMM) programme. The sensitivity and specificity of ICT Malaria Pf® compared to microscopy, as well as factors associated with discordant diagnostic results have been determined in Zambia with 100% sensitivity, 91.5% specificity and 46.7% estimated positive predictive value [17]. Clinical diagnosis refers to cases not diagnosed by either microscopy or RDTs but on the basis of clinical case presentation. Only confirmed malaria cases by either direct microscopy or RDT were included in this study. Case fatality rate (CFR) refers to the proportion of hospital malaria specific mortality of all hospital severe malaria cases. Proportional malaria mortality is the proportion of deaths due to malaria of all health facility deaths.

### Intervention coverage definition

Two exposure variables, IRS coverage and LLINs coverage, were used in this study. IRS coverage was measured as the number of sprayed houses as a proportion of the total targeted houses earmarked for the intervention in a district. As vector control tools, IRS and LLINs are to be deployed at levels of coverage that are high enough (at least 80%) to interrupt malaria transmission [18]. LLINs coverage refers to the number of LLINs distributed as a proportion of the total number of bed nets required to attain universal coverage in a district. Universal coverage of LLINs in the context of Zambia implies covering of all bed spaces (100%) with bed nets [19].

### Study design

Routine surveillance data from the HMIS were analysed retrospectively. Data on malaria in Zambia are relatively complete with over 95% of districts regularly reporting monthly to the HMIS. A desk-based analysis was used to assess the programmatic implementation and epidemiological impact of IRS and ITNs in children below the age of five years between 2007 and 2008. Comparative information on IRS and ITNs was obtained from two published nationally representative cross-sectional population-based household Malaria Indicator Surveys (MIS) conducted in 2006 and 2008 [20,21]. A Demographic Health Survey (DHS) reporting malaria morbidity and mortality and coverage of interventions in 2007 was also used for comparison purposes [22].

### Quality and completeness of data

Routine surveillance data quality and completeness assessment is conducted through district and centrally conducted data audits. The DHMTs hold monthly district information meetings to verify the data before it is submitted to the central level HMIS. The central level clean up the submitted data and any outlying data is verified by following up with the district office. Completeness of reporting is determined based on the proportion of districts that submit data to the central level.

### Sampling

Zambia is divided into 72 administrative districts run by local authorities. The districts are the basic planning levels for health service delivery. Districts were considered as the primary sampling unit (PSU). Routine surveillance data from a total of 30 randomly selected districts were included in the analysis after stratifying by whether IRS or ITNs were the primary vector control interventions. Among these, fifteen districts deployed ITNs and the other fifteen implemented IRS as the frontline malaria transmission interrupting tools. The sample design was taken into account when calculating the confidence intervals. The study monitored the impact of these interventions on confirmed malaria cases, confirmed malaria deaths and case fatality rates in children below the age of five years.

### Statistical analysis

Malaria cases, deaths and case fatality rates in the selected districts were computed for 2007 and 2008. Logistic regression, with population totals and percentage coverage factored into the model to account for between-district variability, was performed to estimate the mean effect of the vector control intervention on malaria cases, proportional malaria mortality and case fatality rates in 2007 compared to 2008. The epidemiological impact of the interventions on malaria cases, proportional malaria mortality and case fatality rates was explored by odds ratios.

## Results

### Routine surveillance data in children <5 years old

Analysis of HMIS data found that the overall absolute number of health facility definitively diagnosed malaria cases reduced by 31% (95% CI = 30–32) from 991,722 in 2007 to 687,396 in 2008, with deaths from malaria reducing by 63% (95% CI = 61–65) from 1,786 to 662 during the same period. The case fatality rates from severe malaria decreased by 62% (95% CI = 61–62) from 35% (95% CI = 34–36) to 23% (95% CI = 22–24 (Table 2). There was substantial inter-district heterogeneity in the number of recorded malaria related deaths and case fatality rates (CFR) across the study period (Tables 3 and 4). The average proportional malaria mortality reduced from 62% (95% CI = 60–64) in 2007 to 44% (95% CI = 42–47) in 2008. Overall, the odds ratio (OR) for 2007 compared to 2008 was 0.5 (95% CI = 0.4-0.5, *P* = 0.082) for deaths and 0.6 (95% CI = 0.5-0.6, *P* = 0.116) for CFR (Table 2) with substantial variations between IRS and ITN districts.

**Table 2:** Rate ratio of malaria cases and odds ratios for proportional malaria mortality in health facilities and for case fatality rates for 2008 relative to 2007, in children < 5 years of age obtained from routine surveillance data in 30 districts, analysed by vector control intervention type in Zambia

**Intervention**	**Proportional malaria mortality in 2007 (n)(95%CI)%**	**Proportional malaria mortality in 2008 (n)(95%CI)%**	**Odds ratio (95% CI)%**	***P***
IRS	63.4 (1990) [61.25-65.49]	38.7 (995) [35.66-41.72]	0.37[0.31-0.43]	0.015
ITN	59.1(889) [55.83-62.29]	54.5 (486) [50.20-58.86]	0.83[0.67-1.04]	0.666
All	62.0(2879) [60.27-63.81]	44.1(1481) [41.54-46.56]	0.48[0.42-0.54]	0.082
Intervention	Cases per 1000 population in 2007 (n)(95%CI)%	Cases per 1000 population in 2008 (n)(95%CI)%	Rate ratio (95% CI)%	*P*
IRS	49.1(1263690) [48.77-49.33]	48.3(939011) [48.20-48.40]	0.97[0.97-0.98]	0.933
ITN	49.9(745447) [49.77-49.99]	49.4(474007) [49.21-49.49]	0.98[0.97-0.99]	0.956
All	49.4(2009137) [49.14-49.58]	48.7(1413018) [48.39-48.91]	0.97[0.97-0.98]	0.944
Intervention	Case Fatality Rates in 2007 (n)(95%CI)%	Case Fatality Rates in 2008 (n)(95%CI)%	Odds ratio (95% CI)%	*P*
IRS	50.3 (24559) [49.71-50.97]	25.8 (15520) [25.10-26.48]	0.34[0.33-0.36]	0.005
ITN	20.0 (26419) [19.55-20.51]	19.3 (14357) [18.64-19.94]	0.96[0.91-1.00]	0.913
All	34.6(50978) [34.22-35.04]	22.7(29877) [22.19-23.13]	0.55[0.54-0.57]	0.116

**Table 3:** **Hospital deaths due to infection with *Plasmodium falciparum *and malaria case fatality rates in children < 5 years of age, observed during routine surveillance in 15 IRS districts in 2007 and 2008 in Zambia**

**Sentinel site**	**% IRS Coverage**	**Hospital malaria deaths as a proportion of all hospital deaths, (%)(n)(95% CI)**		***P *(2007–2008)**	**Case Fatality Rate, (%) (n)(95% CI)**		***P *(2007–2008)**
	**2007**	**2007**	**2008**		**2007**	**2008**	
Chililabombwe	95	45.0(20) [23.20-66.80]	33.3(12) [6.66-60.0]	0.186	13.8(544) [10.89-16.69]	20.4(196) [14.77-26.05]	0.259
Chingola	97	32.6(43) [18.55-46.57]	44.0(25) [24.54-63.46]	0.193	9.6(1446) [8.12-11.14]	15.6(706) [12.90-18.26]	0.232
Chongwe	100	62.5(56) [49.82-75.18]	61.5(13) [35.09-87.99]	0.929	27.8(1260) [25.31-30.25]	19.3(414) [15.52-23.12]	0.216
Kabwe	80	38.6(57) [25.96-51.24]	31.5(92) [22.03-41.01]	0.397	19.6(1123) [17.27-21.91]	30.8(943) [27.80-33.70]	0.115
Kafue	96	40.6(32) [23.61-57.65]	41.9(31) [24.57-59.31]	0.888	14.2(913) [11.97-16.51]	20.6(630) [17.47-23.79]	0.278
Kalulushi	93	27.9(43) [14.50-41.32]	63.6(11) [35.21-92.07]	0.0002	11.5(1045) [9.55-13.41]	10.3(682) [7.98-12.54]	0.797
Kazungula	95	42.9(7) [6.20-79.52]	100(1) […-…]	<0.0001	21.9(137) [14.97-28.83]	28.6(35) [13.60 -43.54]	0.346
Kitwe	100	57.8(36) [36.47-69.09]	46.1(180) [38.83-53.39]	0.251	12.9(1468) [11.16-14.58]	73.6(1127) [71.08-76.22]	<0.0001
Livingstone	94	37.0(54) [24.16-49.92]	16.7(12) [4.42-37.76]	0.0056	48.1(416) [43.28-52.88]	17.7(113) [10.66-24.74]	0.0002
Luanshya	93	50.0(58) [37.13-62.87]	50.0(66) [37.94-62.06]	1	36.6(792) [33.26-39.38]	49.8(663) [45.96-53.58]	0.156
Lusaka	94	64.6(650) [60.94-68.30]	20.7(270) [15.90-25.58]	<0.0001	55.4(2703) [62.91-65.81]	18.2(3075) [16.85-19.57]	<0.0001
Mazabuka	100	38.1(113) [29.10-47.00]	43.2(44) [28.54-57.82]	0.572	16.5(2602) [15.06-17.92]	21.0(905) [18.34-23.64]	0.462
Mufulira	91	31.8(44) [18.06-45.58]	35.2(54) [22.45-47.93]	0.678	18.7(747) [15.94-21.54]	22.8(833) [19.96-25.66]	0.525
Ndola	90	76.6(662) [73.36-79.82]	62.4(157) [54.84-70.00]	0.228	78.4(6468) [77.39-79.39]	25.9(3791) [24.51-27.29]	<0.0001
Solwezi	86	67.8(115) [59.29-76.37]	63.0(27) [44.74-81.18]	0.675	26.9(2895) [25.29-28.53]	12.1(1407) [10.38-13.78]	0.018
All	94	63.4(1990) [61.25-65.49]	38.7(995) [35.66-41.72]	0.015	50.3(24559) [49.71-50.97]	25.8(15520) [25.10-26.48]	0.015

(%) = Malaria deaths as a proportion of all hospital confirmed cases, n=Total number of confirmed hospital cases.

**Table 4:** **Hospital deaths due to infection with *Plasmodium falciparum *and malaria case fatality rates in children < 5 years of age, observed during routine surveillance in 15 ITN districts in 2007 and 2008 in Zambia**

**Sentinel site**	**% ITN Coverage**	**Hospital malaria deaths as a proportion of all hospital deaths, (%)(n)(95% CI)**		***P *(2007–2008)**	**Case Fatality Rate,(%) (n)(95% CI)**		***P *(2007–2008)**
		**2007**	**2008**		**2007**	**2008**	
Chadiza	71	75.6(41) [62.47-88.75]	35.3(17) [12.57-58.01]	0.00013	12.7(2445) [11.40-14.04]	3.7(1604) [2.76-4.60]	0.026
Chama	70.5	67.1(82) [56.90-77.24]	57.8(45) [43.35-72.21]	0.406	14.4(3809) [13.29-15.53]	17.2(1514) [15.27-19.07]	0.619
Chavuma	80	80.0(5) [44.94-115.06]	37.5(8) [3.95-71.05]	0.0009	4.2(942) [2.96-5.54]	10.6(282) [7.04-14.24]	0.096
Chibombo	80	57.9(38) [42.19-73.59]	68.4(19) [47.52-89.32]	0.35	19.7(1115) [17.39-22.09]	21.7(599) [18.40-25.00]	0.756
Chinsali	80	67.7(127) [59.59-75.85]	57.6(33) [40.72-74.44]	0.367	25.5(3379) [24.04-26.98]	20.7(917) [18.10-23.34]	0.48
Kalabo	80	34.7(49) [21.36-48.02]	44.9(49) [30.97-58.83]	0.253	27.1(629) [23.61-30.59]	12.5(1754) [10.99-14.09]	0.020
Kalomo	70.5	50.0(88) [39.55-60.45]	58.7(46) [44.47-72.93]	0.404	48.0(916) [44.79-51.27]	32.4(834) [29.19-35.55]	0.082
Luangwa	100	58.5(41) [43.46-73.62]	22.7(22) [05.22-40.24]	0.00007	43.6(551) [39.42-47.70]	12.6(396) [09.36-15.90]	0.00004
Namwala	80	42.9(35) [26.46-59.26]	72.7(11) [46.41-99.05]	0.006	23.0(653) [19.74-26.20]	47.6(168) [40.07-55.17]	0.0034
Nyimba	75	70.7(92) [61.34-79.96]	55.4(56) [42.32-68.38]	0.173	40.5(1604) [38.12-42.92]	30.7(1010) [27.85-33.53]	0.246
Milengi	75	70.0(10) [04.60-98.40]	83.3(12) [62.24-104.42]	0.283	8.6(815) [06.67-10.51]	18.4(545) [15.10-21.60]	0.059
Mwinilunga	75	60.0(50) [46.42-73.58]	59.6(52) [46.28-72.96]	0.975	10.5(2869) [09.37-11.61]	15.0(2061) [13.45-16.53]	0.373
Samfya	80	51.2(162) [41.07-56.47]	55.6(90) [45.29-65.83]	0.671	24.1(3438) [22.68-25.54]	31.1(1610) [28.86-33.38]	0.346
Sesheke	80	63.0(27) [44.74-81.18]	37.5(16) [13.78-61.22]	0.011	12.3(1385) [10.54-14.00]	44.8(134) [36.36-53.20]	0.00002
Zambezi	80	69.0(42) [55.07-83.03]	74.1(27) [57.54-90.60]	0.0008	15.5(1869) [13.88-17.16]	21.5(929) [18.89-24.17]	0.324
All	95	59.1(889) [55.83-62.29]	54.5(486) [50.20-58.86]	0.666	20.0(26419) [19.55-20.51]	19.3(14357) [18.64-19.94]	0.913

(%) = Malaria deaths as a proportion of all hospital confirmed cases, n=Total number of confirmed hospital cases.

The mean proportional malaria mortality in IRS districts reduced from 63% (95% CI = 61–66) in 2007 to 39% (95% CI = 36–42) in 2008, OR= 0.4 (95% CI = 0.3-0.4, *P* = 0.015) compared with 59% (95% CI = 56–62) to 55% (95% CI = 50–59) for ITN districts, OR= 0.8 (95% CI = 0.7-1.0, *P* = 0.666) (Table 2). The change in proportional malaria mortality was significant in five ITN districts and three IRS districts (Tables 3 and 4).

The average CFR was higher in IRS than ITNs districts (*P* = 0.0003) in 2007, with no significant change observed in 2008 (*P* = 0.333). Considerable heterogeneity was observed in average CFR in IRS districts from 50 (95% CI = 50–51) in 2007 to 26 (95% CI = 25–27) in 2008, OR= 0.3 (95% CI = 0.3-0.4, *P* = 0.005) compared with ITNs from 20% (95% CI = 20–21) to 19 (95% CI = 19–20) respectively (*P* = 0.333), OR= 1.0 (95% CI = 0.9-1.00, *P* = 0.913) (Table 2). In the presence of effective case management through definitive diagnosis and treatment with appropriate ACT, the change in CFR was statistically significant in three ITN districts and four IRS districts (Tables 3 and 4).

here was a positive though not very strong association between the coverage rates of ITNs and IRS and related case fatality rates. The association between ITN coverage and IRS case fatality rate was stronger in 2008 than 2007(Table 2). There was a significant difference between ITN coverage and IRS case fatality rates between the two years (*P* > 0.05). Deaths and cases reduced by 70% (95% CI = 67–72) and by 47% (95% CI = 43–52) in IRS districts and by 27% (95% CI = 26–28) and by 37% (95% CI = 36–38) in ITN districts respectively.

## Discussion

In response to the huge burden of malaria in sub-Saharan Africa [23] and the call by the WHO for scaled-up control efforts [24], coupled with unprecedented availability of resources, targets for malaria control and elimination have been established [25-27]. Attaining these goals require continuous surveillance, monitoring and evaluation of malaria control programmes for adaptation of intervention policy, procedures and methods to optimize the impact of interventions and rationalize resources.

In this study, the average number of malaria cases, proportional malaria mortality and case fatality rates due to malaria in Zambia declined by 31%, 63% and 62% respectively in children less than five years of age. During this period, IRS using pyrethroids and DDT was associated with a significant overall reduction in both proportional malaria mortality and CFR (*P <* 0.05) but the impact of LLINs was not statistically significant (*P >* 0.05). These findings are consistent with those of other studies [28].

While Zambia has made appreciable progress in malaria vector control (Table 1), the observed difference in intervention effect could reflect the challenge of inconsistent bed net utilization [14] and justifies the need for enhanced Information Education and Communication/ Behavioural Change Communication (IEC/BCC) and timely replenishment of worn out LLINs. Despite the difference in efficacy, both IRS and LLINs have had a significant impact on malaria cases, proportional malaria mortality and case fatality rates in Zambia.

The overall reduction in mortality and morbidity observed here cannot exclusively be ascribed to vector control, as ACT was simultaneously being implemented evenly across the country [19,29] and these would have contributed particularly to malaria outcomes but also potentially to transmission [30]. The ACT introduction appears to have resulted in improved treatment seeking behaviour by people and fewer stock outs of anti-malarials in health facilities [31]. There has been improved definitive diagnosis of cases with the roll-out of RDTs [19]. It is plausible that the CFR has reduced (*P <* 0.05) as a result of improvement in case management of severe malaria [28], even if the vector control interventions were of no benefit.

Although other studies have reported impact of combined interventions on morbidity and mortality of all age-groups [28], routine surveillance data have often been considered inadequate for monitoring control programmes [32], and parasite prevalence surveys are most commonly used for assessing impact [17]. Importantly, the reliability of malaria prevalence surveys diminishes with declining prevalence, as the sample size becomes very large [33,34]. While routine surveillance systems have limitations [5], the use of data from both malaria parasite prevalence survey and routine surveillance is important, particularly in areas where parasite rates are below 5% [35,36].

In Zambia, ongoing monitoring of programme delivery and malaria incidence is becoming even more important as the reduced malaria infection rates create zones that are potentially prone to malaria outbreaks [4,35,36]. Such data assists in planning effective response measures. There was marked heterogeneity in the average deaths and case fatality rates recorded in the IRS and ITN areas (*P <* 0.05) and this probably results from inter district heterogeneity in intervention coverage. The malaria control policy striving towards a malaria-free Zambia has facilitated homogenous coverage of integrated malaria control interventions including vector control. This precludes the availability of localities devoid of interventions that could act as control areas since people cannot be denied access to them [5].

In Zambia, routine surveillance data are available across the country and country-wide scaling up of definitive diagnosis using microscopy and RDTs, promotion of IEC/BCC [37], monitoring of the number of laboratory tests undertaken and trends in the malaria (slides or RDT) positivity rate, have assisted in providing more comprehensive data on malaria trends in the country, based on complete HMIS records supported by information from nationally representative household surveys [5,38]. Thus routine surveillance data are a useful resource for monitoring progress and impact of malaria control interventions.

Most malaria control programmes are being monitored and evaluated using clinical and entomological surveys that include parasite prevalence [13,39,40]. This is the first evaluation of the impact of large scale IRS and ITNs on morbidity and mortality in children below the age of five using routine surveillance data at operational population level. The results indicate a marked impact with some variation between the two interventions, although there may well be other important confounders between predominantly rural and urban settings. The decrease in malaria cases, proportional malaria mortality and case fatality rates provide compelling evidence of the reduction of malaria in Zambia following the scaling-up of interventions.

Although there was an overall reduction in deaths and cases in children <5 years of age, there were a number of districts where these indicators remained persistently high. Pin-pointing precisely the factors responsible for persistence of high deaths and cases in these districts could be difficult, as the low impact of LLINs in operational settings could in large part be attributed to waning ownership, use and the physical and insecticide net durability. While high coverage was attained during the scaling-up programme, some nets were distributed as early as 2005. This situation underscores the need for a net replenishment programme and IEC/BCC programme on net use to maintain a high effective coverage [41,42].

The comparatively high impact observed in IRS districts could be as a result of a combination of both IRS and LLINs, as rural parts of these districts may also have received LLINs through the country-wide mass distribution programme. IRS implementation has encroached into rural areas in some districts. In urban and peri-urban areas where IRS is confined, the uptake and utilization of anti-natal and child clinic, and commercially distributed LLINs have also improved markedly in the wake of enhanced IEC/BCC campaigns. This view is further supported by the fact that LLINs coverage in Zambia was similar for the poorest (63%) and richest quintiles (65%) and in urban (59%) and in rural areas (64%) [14].

By April 2009, overall proportional malaria mortality reported from health facilities had declined by 66% in Zambia following scaling up of LLINs and IRS between 2006 and 2008, when proportional malaria mortality declined by 47% and nation-wide surveys showed that parasite prevalence declined by 53% (Table 1). The universal coverage with ITN, IRS and ACT is likely to achieve an even greater decline in malaria burden. In moderate to low transmission setting countries like Zambia, the Roll Back Malaria (RBM) target of reducing global malaria cases by 75% (from 2000 levels) may be attained even several years before 2015 [15] as long as high coverage, as well as effective service delivery, is maintained [43].

This impact assessment was conducted for the period between 2007 and 2008 which may be too short a period to generalize on the observed temporal effects of LLINS and IRS on malaria control. However, the observed reductions in malaria cases, deaths and case fatality rate, in children under-five years of age following scale-up of these interventions are noteworthy findings. As such the use of routine surveillance data in determining the temporal effects of malaria control is an important methodological way forward for malaria monitoring and evaluation.

## Abbreviations

ACT: Artemisinin-based combination therapy; BCC: Behaviour change communication; CFR: Case fatality rate; DDT: Dichloro diphenyl trichloro ethane; DHMT: District health management team; DHS: Demographic health survey; HMM: Home management of malaria; HMIS: Health management information system; HRP: Histidine-rich protein; IEC: Information education and communication; IRS: Indoor residual spraying; LLINs: Long-lasting insecticidal nets; MIS: Malaria indicator survey; NMCC: National Malaria Control Centre; PSU: Primary sampling unit; RBM: Roll Back Malaria; RDT: Rapid diagnostic test; WHO: World Health Organization.

## Competing interests

The authors declare that they have no competing interests.

## Authors’ contribution

EC: Co-designed the study, collected and analysed the data, and drafted the manuscript. MarlizeC guided in data analysis and interpretation and contributed to the drafting of the manuscript and critically evaluated it. IK and BH: Assisted with data analysis and interpretation and were involved in the drafting of the manuscript. FM and PCK: Participated in data interpretation and drafting of the manuscript. MC, DND, JH, and KSB: Critically reviewed the manuscript. All authors read and approved the final manuscript.
